# Acknowledgments

**Published:** 1859-01

**Authors:** 


### ACKNOWLEDGMENTS.

We are much obliged to the Esculapian Society for the generous remittance received from their secretary. We shall ever take pleasure in giving publicity to such portions of their transactions as they shall see fit to send us.

As the present number commences a new volume, we hope all our friends will remember it as a favorable time to commence their subscriptions. We shall do all in our power to make the present volume decidedly more valuable than any of the preceding ones.

